# Mr. Robertson on the Health of English Manufacturers

**Published:** 1832-01-01

**Authors:** 


### General Remarks on the Health of English Manufacturers; and on the Need for Convalescent Retreats, &c. By Mr. *John Roberton*, of Manchester. Octavo, pp. 36.

Mr. Roberton's pamphlet consists of three letters, two of which were published in the Manchester Guardian, while the third, to which the others are introductory, is addressed to the candid and deliberate consideration of his professional brethren—as well as to all those who take an interest in the well-being of that vast and important class of society—the operative manufacturers.

In a late work by Mr. Senior, entitled "Lectures on the Rate of Wages," the following passage appears:—

"'The Englishman's industry may sometimes be excessive, his desire to better his condition may sometimes drive him to toils *productive of disease*, ill recompensed by the increase of his wages; but that such *is not generally* the case, may be proved by comparing the present duration of life in England with its former duration, or with its duration in other countries.' It is further stated that the average duration of life appears to have much increased within the last fifty years; that the average mortality amongst savages is the greatest that is known; that on the continent the mortality is *one* in *thirty-four* of the inhabitants yearly; that in England, about a century ago, when more than one-half of the population was agricultural, it was supposed to be *one* in *thirty;* fifty years ago, *one* in *forty;* thirty years ago, *one* in *fifty-four;* and now, when two-thirds of the people are manufacturers, and more than one-third dwell in cities, it is *one* in *fifty-eight.*" 5.

Mr. Roberton doubts the conclusion to which Mr. S. and other political writers have come; namely, "that the greater or less prevalence of disease in a population must *necessarily* regulate the duration of life—or, in other words, that where the duration of life is on the increase, *there* an increasing diffusion of health may be predicated, and *vice versa.*" We are quite persuaded that this conclusion is erroneous, and the reverse is nearer the truth. The vulgar opinion even goes to the length that valetudinarians are proverbially long-lived—and that robust health is far more liable to those acute and dangerous diseases that either snap the thread of life, or lay the foundation for organic changes in the vital organs. Life is, no doubt, shorter among barbarians than among civilized people; but this is evidently not from their being feeble or sickly. Their ignorance of the causes of diseases, as well as of the treatment—their liability to famines and epidemic influences—their ruthless wars—and lastly, the waste of life from abortion, infanticide, and infantile mortality, must be taken into account in the comparative shortness of life among savage nations. Our author is of opinion that, if the matter could be fairly ascertained, it would be found that English manufacturers, although they enjoy a longer life than barbarians, yet that they pass a greater proportion of their time in a state of disorder and suffering. To savages, death generally comes as a sudden stroke, in the form of some acute disease, as fever, dysentery, &c.—or in the shape of war. Few indeed are the subjects of chronic maladies. Their constitutions are good, and they will recover rapidly from wounds which would kill Europeans in a short time. Upon the whole, we may safely conclude that the ruder tribes of mankind, in compensation for want of intellectual enjoyment, possess a considerable and uniform degree of animal vigour—and that, while their days are shorter in number, they are more exempt from bodily suffering than those who compose our great manufacturing communities.

"The hitherto increasing duration of life in England is no disproof of this latter remark. It is to be accounted for, among other causes, by the extraordinary improvements which have taken place in medicine, and all its collateral branches, within the last eighty years; by the gratuitous medical aid now almost universally afforded to the poor, which places them, in this most important particular, on a level with the rich; and, not least, by the increase which has taken place in the means of subsistence—a circumstance that has been singularly favourable to the rearing of healthy children. Indeed, it is probable that the difference which exists in regard to *the duration* of life in this country and among barbarians, is owing, chiefly, to the far greater chance of life in infancy, with us, than with the latter. Moreover, there are various peculiar causes which have tended to lengthen the duration of life in England, without producing a correspondent exemption from disease. A century and a half ago, violent epidemics, such as fluxes, agues, spotted fevers, and small-pox, as well as inflammatory diseases, were far more common than they are now, and incomparably more fatal. In place of these, however, chronic affections, (many of which are compatible with considerable longevity,) originating in the head, chest, abdomen, and pelvis, owing to the artificial life which two-thirds of our population are compelled to lead, have greatly increased; and in thousands of instances, in every considerable town, render existence one long disease." 12.

The number of dispensary patients in Manchester demonstrates that a very large proportion of our operative population is annually on the sick-list. The town in question contains 227,000 inhabitants—and during the year 1830, which was by no means sickly, the home and out patients of the four great general dispensaries, amounted to 22,626—while at least ten thousand more received relief from several other institutions, as the Eye-Infirmary, Children's Dispensary, &c. &c. If to this immense list we add the private patients prescribed for by the regular faculty—by quacks—by chemists, &c. we may be safe in concluding that *three-fourths* of the inhabitants of Manchester are annually necessitated to submit to medical treatment!

The sedentary and other avocations which wholly seclude the artisan, at all seasons, from the open and pure air, unquestionably give rise to derangements of the general health, manifested primarily in the functions of the digestive organs, rendering the mind torpid or irritable. These morbid conditions are aggravated too often by the means adopted to obtain relief—as the drunkenness—the excitement of politics—of riot and uproar—and unhappily of " savage or malignant crime." Our author is convinced that, numerically, " the middle class does not bear so high a proportion to the lower class as it did half a century ago."

" And with regard to the actual condition of the latter, which now composes so overwhelming a proportion of our population, it is, in several respects, in a worse condition than at any period within the last two centuries. At least, I think it might be shown that they are less healthy, according to my definition of health, and more depraved and mal-content than perhaps at any former period." 15.

Our author observes that the science of political economy appears to refer entirely to wealth, without any eye to the happiness of those who produce it.

" Judging from much which I see continually occurring around me in society, I should be inclined to say that as the production of wealth is obviously retarded by vice, ignorance, and discontent, and promoted by morality and social happiness, political economy ought to be regarded as essentially a branch of ethics. How greatly, for example, is the productiveness of the labour of a manufacturer lessened, by his spending (as so many of his class do) one or two days weekly in the alehouse! Again, in the numerous long-continued suspensions of production, occasioned by disputes between masters and their workmen, how much wealth is lost, which a different moral and intellectual condition of the parties would have secured to the country! It would be impertinent were I to enlarge upon this topic in a note: many other illustrations of the truth of my remarks will occur to every one practically conversant with the condition of our manufacturing population. I should suggest, as an improvement on Mr. Senior's definition of political economy, that it be defined as—*The science which treats of the promotion of the physical and moral welfare of the members of a community, by means of the production and distribution of wealth.*" 16.

Pursuing the subject of his investigation in Letter the second, our author defines health to be " that condition of the body, in which the organic and animal functions are performed *with a feeling of satisfaction.*" We never quarrel much about definitions: but we apprehend that, as far as regards the *organic* functions, there is no *feeling* of any kind in perfect health. Surely a man ought not to *feel satisfactorily* the circulation of his blood, the digestion and assimilation of his food, the secretion of bile, &c. These operations ought to go on insensibly. We are ready to grant that, in regard to the animal functions, as the exercise of the limbs, the different senses, and the appetites, there is a feeling of great satisfaction when in perfect health —and *vice versâ* in disorder. There is, we fear, a very large proportion of our manufacturing population in a condition of health, where the various functions of mind and body are carried on with little feeling of satisfaction, but with much feeling of discomfort.

" Undoubtedly the hardest part of the operative's lot consists in his being so very liable to disease, without at the same time possessing the power of relaxing from his labour. Day after day, the mule or the loom moves the accustomed number of hours, and the full amount of work must be done. There is not, and in the present state of things there cannot be, two grades of labour—one for the whole, and another for the sick. The consequence is, that hundreds of instances of indisposition, which, at the beginning, might soon have been removed by repose and relaxation, become, owing solely to the want of these, confirmed and more and more inveterate, till the stock of vigour is entirely exhausted, and the health ruined; or in such a state as to require months for its re-establishment —an alternative fraught with the utmost pecuniary difficulty in many cases, and the want of proper cordials, nourishment, and the requisite change of air, in nearly all." 21.

These hardships must inevitably press harder upon the artisan than upon any other class—for instance, the agricultural labourer—for obvious reasons. It is, our author remarks, a great misfortune for an operative family to become resident in a densely-peopled community like Manchester, where the working classes are, numerically, to the upper classes, who live within the limits of the town, as ten to one. Hence there are whole districts, in which there is hardly an individual to whose superior habits and manners they can look up to as a proper example—or upon whose more enlightened advice and sympathy they can depend in their domestic exigencies. It is owing to this state of society, that every kind of vice multiplies itself with astonishing rapidity.

"When, in every second or third dwelling, we may find a drunkard, a profane or an obscene person, (and in many districts such characters are even more plentiful than this) who can escape the influence of evil example? When vice is daily (and nightly too) familiar to every eye and ear, what but a miracle can prevent general corruption? *Here* are to be seen early profligacy, contempt of parents, improvident marriages, neglect of religion—even to utter heathenism, insubordination to superiors, the most sluttish waste, dishonesty, general tippling in both sexes, pauperism, gloomy discontent, and the frequent occurrence of disease. These are the circumstances, surrounded by which a well-disposed operative has to encounter the ordinary difficulties of his lot." 23.

What has been said of districts is applicable to large manufactories, where 800 or 1000 operatives are crowded together, and superintended by a single employer. Here there can be no gradation of rank, so useful in society. The master has no bond between him and his labourers. He does not even know their names or faces—has nothing to do with their conduct, except *as manufacturers*—and, in fine, cannot do otherwise than keep entirely aloof from them:—Hence must originate a condition of mind, in the operatives, at once low, conceited, and insolent—"a very hot-bed for turbulence and crime." Further, we have to deplore the necessity which the operative is under of labouring so many hours daily in bad air, and at an exhausting occupation. After the toils of such a day, how is the sinking and uneducated mind to obtain a comfortable sense of existence—but by means of inebriating liquors? In such a family, all instruction of children and economical domestic arrangements are neglected. Sunday-schools offer a very inefficient remedy! The present state of our great manufacturing population has evidently not improved their health; for the annual mortality at Manchester is 1 in 45, while that of England generally is 1 in 58. We may, however, console ourselves with this reflection, that the annual mortality in Naples, the finest climate of Italy, is just double that of Manchester, according to the bills of mortality in 1830!

In the third Letter, our author, disclaiming all attempts to point out moral or political remedies for the evils alluded to, directs his attention to a physical remedy—"the means best calculated to counteract the effects of protracted deviations from health, so prevalent in our population." He confines his remarks and suggestions to the inhabitants of Manchester; but it is obvious that whatever means of amelioration are applicable to them, will be so to the population of all other great manufacturing towns.

To our author, then, it has appeared, that a great proportion of those patients who apply at the public charities for medical assistance are in a condition to derive little or even no benefit from the most skilful medical treatment. They are labouring under some form of chronic disease, which, in its nature, may or may not be incurable—and, while under treatment, are generally compelled to pursue their ordinary avocations—their residence, in a majority of cases, being a cellar—their food of the worst description. It is hardly necessary to observe, that such patients require *something* besides medicine! They are more in need of a removal from the influence of those depressing causes, which, if not the sole origin of their complaints, have tended to aggravate and increase them—and a continuance of which must protract, if not baffle, all attempts to cure. The following illustration will apply to more places than Manchester or Birmingham.

"A man I shall suppose, (and I choose a familiar illustration,) of delicate constitution, of sedentary occupation, residing in a confined situation, and who has to provide for a large family, becomes the subject of chronic dyspepsia. Anxiety, unsuitable diet, unremitting labour, combined with the want of even the occasional inhalation of a pure atmosphere, tend to aggravate the disease. He is admitted on a Dispensary. His sallow, desponding countenance, and other prominent symptoms, speedily reveal the nature of his malady. He is forthwith put under the most judicious treatment. For a time he appears to have obtained relief. The alterative has perhaps corrected the vitiated state of the excretions: the prussic acid, or the strychnus, has eased the pain of the stomach; but these indications of amendment are of short continuance. After a few weeks they are vanished. The medicines are now varied; and new hope is held out; but 'the bloom of hope' is gone. The patient returns to his last, his needle, or his loom; and no less to his cares, long hours, close confinement, and vegetable diet; and in due time his name appears on the Dispensary Register, as one of the 'Irregular;' an appellation which has the merit of saving the credit of the physician, by throwing the blame of failure on the patient. In this case it will surely be admitted that *something* besides medicine is wanted." 28.

The following is a striking and true picture of the almost miraculous effects of pure air, change of scene, and temporary change of condition, in ill health.

The mother of a family has a miscarriage, attended with hæmorrhage, which leaves her in a state of abject weakness, a prey to a train of nervous symptoms. What is the fate of this poor woman? She attends at the Dispensary, week after week, with a straw-coloured melancholy visage, a hurried circulation, headach, feeble digestion, swelled ancles, and other indications of debility. The surgeon does his best, but he knows that his patient, notwithstanding her weakness, has daily to undergo the drudgery of doing, single handed, for her family; that her residence is a cellar, or some other equally unpropitious dwelling in a confined situation; that her diet, which ought to be better than common, is the ordinary diet of a poor family, and altogether unsuitable to the sickly appetite of such an invalid. After long-continued, persevering, but fruitless attempts to benefit his patient, the surgeon is fortunate enough, (such fortune, I may remark, is rare,) to obtain for her a Southport-charity ticket, with which she is immediately dispatched to the watering-place of that name, for the prescribed term of three weeks. Here a *total change* in her circumstances takes place. She breathes a pure bracing atmosphere; has clean airy lodgings; plenty of wholesome food; and no other duties to perform than those arising from the care of her person and her daily walks. And what is the effect? Her appetite and spirits return; (begin to return perhaps the very day she arrives;) and when, on the expiration of her term, she returns to her surgeon, he finds her perfectly, or nearly, recovered; and gladly admits that in such forms of disease as this, coupled with the already-mentioned adverse circumstances of a personal kind, a *temporary change* of *condition*, such as this removal produces, has incomparably more influence in the restoration of health than the most skilful medical treatment has in thrice the time without it." 29.

The author proposes a remedy, as far, at least, as his own town is concerned. It appears that, for a number of years past, there has existed a "stranger's bathing charity" at Southport, supported by a public subscription, and which, though limited in its means, affords relief annually, to a good many poor affected with scrofulous and other complaints supposed to be benefited by sea-air and sea-bathing. The plan which our author proposes is somewhat different from the one in question, and we shall lay it before our readers.

"I propose first, that an Institution be founded, (to be named a Retreat for Convalescents,) in some salubrious, and otherwise eligible situation, at a distance from town, and if possible on the sea-coast, for the reception of such patients of our Medical Charities, as the committees of these Charities may recommend as fit objects.

Secondly, that the convalescents shall be accommodated in a suitable building, to be under the superintendence of a responsible married couple, who shall officiate as governor and matron respectively, of the establishment.

Thirdly, that the expenditure of the Retreat (I am assuming that the buildings, &c. have been erected by public contributions:) shall be sustained by annual subscriptions chiefly, not omitting other means of income: the number of convalescents admitted annually to maintain a strict relation to the income of the current year, whatever that may be.

Fourthly, that the Retreat shall be open for patients, during eight months of the year, namely, from the first of April to the thirty-first of October.

Fifthly, that a regularly qualified apothecary, resident in the neighbourhood, shall be appointed to the Retreat, whose services shall, if possible, be obtained gratis.

From known data, I am warranted in assuming, that a clear income of twelve hundred pounds per annum, would allow of at least one thousand patients spending each three weeks at the Retreat, in the enjoyment of plain wholesome food. And further, that for the accommodation of this number, about fifty beds would be required, with half a dozen spare beds, in case of accidental sickness; it being understood that no persons labouring under active forms of disease, should on any account find admission." 34.

The author very properly reminds the wealthy inhabitants of these isles of the numerous convalescent retreats, scattered along the coasts and throughout the interior of the country. The whole line, indeed, of the English coast, is thickly studded with watering-places that have been called into existence for their accommodation; while Bath, Buxton, Cheltenham, Tunbridge Wells, and fifty other great Houses of Recovery afford sources of amusement and health to the affluent of these isles. We applaud Mr. Roberton's benevolent designs, and cannot but sincerely approve of his proposals. If the great lords of the creation were to dedicate a portion—even a small portion—of their enormous revenues to the restoration of health among the humble classes of "unwashed artisans," instead of squandering their fortunes in foreign countries, encouraging Paganinis and other exotic parasites, they would do more good to their country—and perhaps ultimately to themselves, than they now do! But we fear the still small voice of humanity will seldom reach their ears—unless they should be opened, when too late, by the thunder of some national convulsion.

